# Brazil is already experiencing the brutal impacts of climate change

**DOI:** 10.11606/s1518-8787.2026060007009

**Published:** 2026-02-13

**Authors:** Camila Lorenz, Thais Araújo Cavendish, Thiago Salomão de Azevedo, Michelle Bell, Adelaide Nardocci, Maria de Fátima Andrade, Thiago Nogueira

**Affiliations:** I Universidade de São Paulo. Faculdade de Saúde Pública. Departamento de Saúde Ambiental. São Paulo, SP, Brasil; II Instituto Butantan. Departamento de Parasitologia. São Paulo, SP, Brasil; III Universidade de São Paulo. Faculdade de Saúde Pública. Programa de Pós-Graduação em Saúde Global e Sustentabilidade. São Paulo, SP, Brasil; IV Secretaria Municipal de Saúde de Santa Bárbara d'Oeste. Santa Bárbara d'Oeste, SP, Brasil; V Universidade Estadual Paulista. Instituto de Biociências. Departamento de Biodiversidade. Rio Claro, SP, Brasil; VI Yale University. School of Forestry & Environmental Studies. New Haven, CT, United States; VII Universidade de São Paulo. Instituto de Astronomia, Geofísica e Ciências Atmosféricas. São Paulo, SP, Brasil

**Keywords:** Climate Change, Disasters, Disaster Mitigation

## Abstract

Brazil is increasingly experiencing severe climate events, including extreme droughts, wildfires, floods, and heatwaves, driven by both excessive rainfall and prolonged dry periods. These disasters have resulted in significant environmental, economic, and social losses, deepening inequality and fuelling public health crises. Climate change is disproportionately affecting vulnerable populations and contributing to the rise of disease outbreaks such as dengue and Oropouche fever. Brazil's vulnerability stems from its diverse ecosystems, heavy reliance on agriculture and hydropower, and its critical role in global climate dynamics due to widespread deforestation. This paper examines the country's future challenges and outlines strategies to address extreme weather events, including the development of climate adaptation policies, enhanced deforestation monitoring, and strengthened disaster preparedness. To improve resilience, Brazil must invest in comprehensive risk assessments, the integration of disaster risk indicators, and the establishment of a national climate-disaster reporting system to better anticipate, mitigate, and manage the impacts of extreme climate events.

## INTRODUCTION

Recently, extreme weather and climate events in Brazil have exceeded the country's historical averages. The number of these events (flooding and heatwaves) is related to the country's vast size, with a significant portion of its territory located in tropical regions. Approximately 85% of Brazilian climatic disasters are linked to excessive rainfall or its absence^1^. During the severe 2020 drought, the Pantanal biome endured its most devastating fire outbreak, engulfing hundreds of thousands of hectares in flames, endangering habitats and vital ecosystem functions. Fires encroached on areas that had never or rarely experienced such devastation, including national conservation units^2^. In northern Brazil, an unprecedented heat wave struck the Amazon in July 2023, exacerbating one of the most severe droughts recorded. According to Port of Manaus officials, the water level of Rio Negro dropped to 12.70 meters in October 2023, marking the lowest point ever recorded since observations began in 1902^3^. In the southern region, rapid river flooding and overflow of Lake Guaíba in 2024 inundated numerous cities in the state of Rio Grande do Sul (Figure), marking the country's most significant environmental catastrophe to date^4^. Moreover, 2023 was the hottest year on record in Latin America. Heatwaves affected health throughout the year and elevated mortality rates. This increased temperature could have also caused the significant surge in dengue cases in the region, with cases more than tripling compared with 2023^5^. Temperature plays a crucial role in both the virus's activity and behaviour of mosquito vectors.

**Figure:**
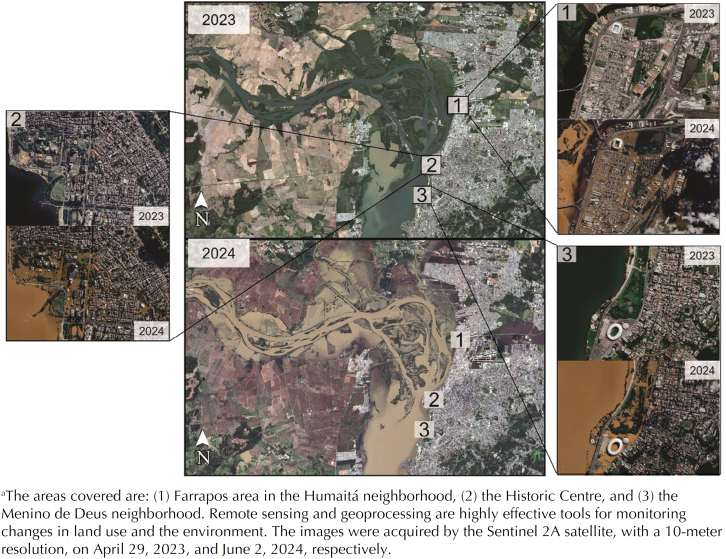
The State of Rio Grande do Sul, Brazil, before and after the largest flood in the country's history in April 2024^a^.

Drought-related disasters cause substantial economic and social loss. From 1970 to 2019, natural hazards such as floods, landslides, and droughts caused damage amounting to R$ 250 billion (approximately US$ 42.0 billion) in Brazil^6^ and resulting in over 10 thousand deaths over the past five decades^7^. However, these economic projections might be an underestimate, as they fail to account for the complete impact on health. From 2000 to 2018, nearly 48,000 deaths were attributed to the increasing heat waves in the 14 most populous Brazilian metropolitan regions, and higher mortality rates were observed for people with low educational levels, people of colour, older adults, and women^8^. Additionally, changes in rainfall patterns and temperatures can transform new areas into hotspots for disease transmission via water or animal vectors^9^, such as the ongoing Oropouche fever outbreak^10^. In 2024, the virus was detected in urban/rural areas where no transmission had previously been reported.

### Challenges in the Brazilian Scenario

According to the latest Intergovernmental Panel on Climate Change (IPCC) report^11^, a climate collapse is imminent without intervention. Northern Brazil is expected to become cooler and drier, potentially disrupting the Amazonian ecosystem. Similarly, the already-arid Northeast will experience intensified dryness, severely impacting water, energy, and food security. Heat and dryness will also increase in the central-western region, a Brazilian agribusiness hub. Additionally, the southeast is projected to become hotter and experience water-related climate extremes more frequently. The recent river flooding in southern Brazil, which submerged several cities in the state of Rio Grande do Sul^12^, serves as a stark warning that Brazil is already experiencing the severe effects of climate change. Additionally, Brazil faces extreme social and economic inequality, and these events further exacerbate existing disparities. Recently, the country has experienced significant setbacks in environmental protection and public health policies^13^, which have been worsened by the pandemic and scientific denial. As Brazil's environmental regulations were dismantled, attacks on science put pressure on local efforts to combat deforestation and exacerbated long-standing inequalities and injustices in Brazilian society. Brazil confronts numerous challenges, including integrating extreme climatic events into the public health agenda, strengthening strategies to tackle deforestation, and urgently developing climate adaptation policies. As the frequency and severity of excessive rainfall and drought are likely to intensify, effective monitoring, forecasting, and impact assessments should support mitigation and preparedness actions. In a vast country such as Brazil, evaluating drought impacts is crucial because of the diverse vegetation, soil types, land use, and varying climate regimes. The Brazilian Interministerial Committee on Climate Change is now preparing a Climate Adaptation Plan within the scope of the new Climate Plan 2024–2040 to strengthen the country's resilience and promote climate justice^14^. Since 2009, with the National Climate Change Policy, the federal government has formulated National and Sectoral Adaptation Plans; however, the true effectiveness of these instruments is not yet understood. The health sector has advanced with the development of adaptation, contingency, and response plans for extreme climate events, with an assessment of vulnerabilities and governance mechanisms for climate and health policies, but preparedness and response capabilities are lacking^15^. Healthcare facilities in Brazil are severely affected by climate-related disasters, abruptly interrupting services and generating increased public spending^16^.

### Recommendations for an Integrated Climate and Health Agenda

Here we discuss key research priorities for better understanding extreme climatic risks for Brazil. Accurately addressing these research gaps is crucial for better assessing the risks of both excessive rainfall and drought, and for developing public health policies that effectively mitigate their impact.

#### Identification of At-Risk Areas and Populations

Clear definitions of risk and a thorough understanding of how to incorporate its components into a comprehensive analysis are crucial. Identifying key factors of risk in assessment methods enables the prioritization of resources for the most vulnerable areas and populations. Omitting any component can lead to biased and inaccurate risk estimates, resulting in poor resource allocation, failure to identify critical areas, and prolonged exposure of vulnerable populations to unmanaged risks. However, due to the complex, interconnected social, economic, cultural, and environmental systems, capturing the full system remains challenging. A gap in drought research can be found in the regional or spatial patterns of droughts. Similar to regional flood frequency analysis, there is a need for "regional drought frequency analyses." Understanding the spatial extent of drought duration, severity, and/or intensity is crucial for planning effective measures to mitigate drought impacts^17^. According to Alcântara et al.^12^, urban expansion has led to deforestation, soil sealing, and an increase in impermeable surfaces like roads and buildings. These changes interfere with natural drainage systems and raise surface runoff, which in turn heightens the risk of urban flooding. Furthermore, research points out that informal settlements—often built in high-risk areas with poor infrastructure—further increase vulnerability to extreme weather events^12^. A recent development in this field is the Google Earth Engine platform and its spatial analysis capabilities, which have opened up new possibilities for processing and analyzing large-scale data quickly. The platform offers access to over 40 years of climate data for virtually every location on the planet^18^. This process requires an interdisciplinary approach, involving collaboration across public health, ecology, climatology, geology, and socioeconomics. Such integration is essential for tackling the complexities of climate change and ensuring a coordinated and effective response to emerging disasters.

#### Enhancing Risk Models and Uncertainty Analysis

Currently, assessing risks associated with extreme climatic events relies heavily on the expertise of modelers and the limitations of available data. However, when multiple models address the same issue, comparing their results quantitatively can be challenging due to uncertainties in both the models and the data. To overcome this challenge, there has been progress toward establishing clear conceptual frameworks and promoting standardized approaches. To develop comprehensive risk models, it is essential to clearly define the methods for analyzing these factors.

For instance, when evaluating local flood risk, various aspects of human health and the environment must be considered. Spatial layers representing land use help assess hazard levels, while factors such as population density and the incidence of diseases like leptospirosis provide insights into exposure. Additionally, vulnerability assessments might examine the proximity of communities to healthcare facilities. Integrating these factors into a unified model allows for a more precise representation of overall risk.

Statistical methods that address the duration of droughts and floods are fairly advanced, but techniques focused on severity are less developed and need significant enhancement and refinement^17^. There is a clear need to create methods for predicting the onset and/or cessation of droughts and floods. Approaches such as pattern recognition, physically-based models, a moisture adequacy index using Markov chains, or the concept of conditional probability appear promising for developing reliable and robust forecasting methods to achieve this objective^17^.

Despite following a common framework, each risk assessment is uniquely influenced by the specifics of the extreme event, the available data, and the geographical and temporal context of data collection. Risk analysis models should incorporate estimates of model uncertainty, and routine data collection efforts are necessary to enhance public health preparedness for future extreme climatic events.

#### Integration of Risk Indicators Into Monitoring Frameworks

Adopting a Health in All Policies (HiAP)^19^ approach can help develop more cohesive and effective policies that address the multifaceted nature of extreme climatic event risks. As a practical and systematic approach, HiAP relies on cross-sectoral collaboration to develop frameworks and action plans grounded in a holistic understanding of how policies across sectors influence health determinants^19^. Incorporating various indicators for disaster risk assessment into monitoring frameworks and action plans can enhance our understanding and integration of health considerations across different policy agendas. For example, in areas more prone to flooding, the risk of water-borne diseases such as cholera, diarrhea, and leptospirosis should be taken into account. In regions where drought is more common, the storage of clean water in containers can lead to increased breeding of *Aedes aegypti* mosquitoes, potentially resulting in a rise in dengue cases. All these interactions must be considered when developing disaster and health indicators. Moreover, implementing this approach offers valuable insights into evaluating the effectiveness of integrated mitigation and health programs. By employing rigorous research methods, such as impact evaluations, we can assess the causal impact of these programs on both environmental and health outcomes. This evidence-based approach enables us to identify effective strategies for mitigating extreme climatic event risks and enhancing overall public health resilience.

#### Strengthening the Brazilian Unified Health System

The health sector plays a crucial role in supporting and enhancing disaster management to prevent morbidity, mortality, and health risks. This is particularly important for actions aimed at reducing both the direct impacts of extreme events (such as deaths and disabilities) and their indirect consequences (such as damage to infrastructure and living conditions). Climate emergencies, disasters, and public health crises are connected with broader environmental, health, social, and political challenges, necessitating enhanced organizational and response capacities from the Brazilian SUS^20^. In the short, medium and long term, SUS faces several challenges, especially due to the need for more resources and the optimization of the use of public funds. It is essential to employ community health workers to deliver healthcare services to rural and vulnerable populations, while also providing training for them to serve as first responders and health educators, especially in remote areas with limited access to healthcare. Additionally, it is crucial to establish continuous and thorough training programs that emphasize disaster preparedness, climate change awareness, and health risks linked to climate change, such as managing the rise in disease prevalence due to climate variability^21^. Health professionals must receive disaster-focused training that includes hands-on drills, theoretical lessons, and policy analysis activities tailored to the specific climate risks of the local area.

#### Creating an Integrated Climate-Disaster Platform in Brazil

At present in Brazil, there is no national database that comprehensively collects extreme event risks and other climate-related data from health systems or monitors the achievement of targets. To effectively oversee the national scenario, new or significantly enhanced infrastructure and data platforms are required.

Several countries maintain national platforms dedicated to monitoring and reporting climate-related disasters, with varying levels of integration between meteorological data, social impacts, and institutional responses. In the United States, the National Oceanic and Atmospheric Administration's (NOAA) system — especially the Storm Events Database and the Billion-Dollar Weather and Climate Disasters tracker^22^—offers detailed historical series of extreme events, including estimates of economic damage and human losses. Australia operates the Disaster Mapper^23^, which combines local resilience knowledge with accessible records of events, response strategies, and recovery efforts. In Germany, the national meteorological service works alongside the Federal Office of Civil Protection and Disaster Assistance to map natural hazards, highlighting coordination between science and civil defense^24^. In Brazil, the National Center for Monitoring and Early Warning of Natural Disasters (CEMADEN) is a significant initiative in tracking hydrometeorological risks, providing real-time alerts and geospatial monitoring of events such as floods and landslides^25^. However, the country still lacks an integrated platform that combines disaster data with epidemiological, socioeconomic, and public health information. To move forward, it is essential to connect CEMADEN's data with systems from the public health service (SUS), civil defense, and the *Instituto Brasileiro de Geografia e Estatística* (Brazilian Institute of Geography and Statistics), in order to build composite indicators that reflect not only the occurrence of disasters but also their impacts on vulnerable populations—thereby strengthening response and prevention efforts in the context of climate change.

## CONCLUSION

Droughts and floods are often viewed as rare, extreme events in climate systems, but, in reality, they should be understood as regular occurrences, particularly in the context of climate change. They have happened repeatedly in the past and will continue to occur; however, their negative impacts are expected to grow in the future. As a result, the impacts of drought and floods should be managed through a risk-based approach rather than the crisis-driven approach currently employed in most countries. This approach requires a centralized database, a national reporting structure, and clear strategies supported by administrative and informatics governance.

To address the growing challenges posed by climate change, it is essential to accurately identify the areas and populations most at risk of disasters. Risk models must clearly define the factors contributing to vulnerability, guiding both policy development and the prioritization of health and social protection actions. Integrating disaster risk indicators with ongoing monitoring frameworks is crucial to ensure more effective, timely, and evidence-based responses. In this context, strengthening the SUS is a strategic measure—not only to ensure care for affected populations, but also to coordinate epidemiological, social, and environmental information at the national level. Establishing a national climate-disaster reporting and monitoring platform that brings together these multiple systems and sources of knowledge represents a critical step toward enhancing the country's ability to prevent, respond to, and adapt to climate impacts with equity and efficiency.

Researchers play a critical role in shaping these frameworks, which need to be considered by policymakers at both national and international levels and implemented by local practitioners. Addressing governance challenges, including both vertical and horizontal integration, is essential to foster meaningful discussions and actions to mitigate flood and drought impacts in urban areas. Policymakers must adopt comprehensive and aligned strategies that incorporate recommendations for managing floods and droughts, avoiding fragmented responses across national, regional, and local levels, and ensuring a more holistic approach to tackling these interconnected hazards.

## Data Availability

All the data supporting the results of this study were published in the article itself.
